# Monitoring Key Parameters in Bioprocesses Using Near-Infrared Technology

**DOI:** 10.3390/s141018941

**Published:** 2014-10-13

**Authors:** Elena Tamburini, Maria Gabriella Marchetti, Paola Pedrini

**Affiliations:** Department of Life Science and Biotecnology, University of Ferrara, Via L. Borsari, 46 I-44121, Italy; E-Mails: mch@unife.it (M.G.M.); pdp@unife.it (P.P.)

**Keywords:** near-infrared spectroscopy, fermentation, on-line monitoring, in-line monitoring, calibration curves, bioprocess control

## Abstract

Near-infrared spectroscopy (NIRS) is known to be a rapid and non-destructive technique for process monitoring. Bioprocesses are usually complex, from both the chemical (ill-defined medium composition) and physical (multiphase matrix) aspects, which poses an additional challenge to the development of robust calibrations. We investigated the use of NIRS for on-line and in-line monitoring of cell, substrate and product concentrations, during aerobic and anaerobic bacterial fermentations, in different fermentation strategies. Calibration models were built up, then validated and used for the automated control of fermentation processes. The capability of NIR in-line to discriminate among differently shaped bacteria was tested.

## 1. Introduction

Development and optimization of bioprocesses are strongly dependent on accurate, real-time control of chemical and physical process variables, for increased productivity, efficiency and reproducibility. Established sensor technologies for some of the key parameters that have an influence on the process, including temperature, pH and dissolved oxygen, are already in common use for process control [1,2].

However, research is still underway on sensors systems for other critical variables, as medium composition, biomass concentration, product concentration, and the metabolic state of the cells [3].

Moreover, this potentially large number of chemical parameters are desirable to be measured simultaneously and in real time. Approaches based on specific sensors, while solving specific analytical problems, do not address this basic requirement of bioprocess monitoring and control. Moreover, all of the methods based on traditional instrumental chemistry (*i.e.*, chromatography) require that a sample be withdrawn from the bioreactor and extracted, centrifuged and pretreated before the analysis. This means that there is an unavoidably time delay in the process data acquisition, and often, recurring sample withdrawals cause sterility problems [4].

In practice, techniques suitable to acquire biochemical data from fermentation processes should ideally enable one to:

– maintain the asepsis of the process;
– be selective to exclude interference by other process variables;
– be insensitive to changing process conditions;
– possess a wide detection range to cover changes during the course of the process;
– produce a fast response to enable corrective action to be taken;
– be stable to minimize the need for re-calibration;
– require little maintenance;
– guarantee a short return of investments.

While some of these factors are particularly important in the monitoring of industrial processes, namely the maintenance and cost aspects, the rest are equally important in the knowledge acquisition process, leading to the development of process models, also at the lab scale [5].

**Figure 1.**
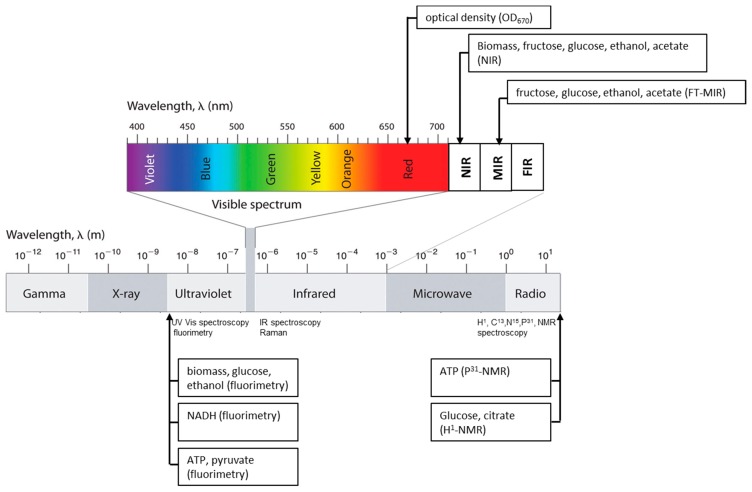
Current spectroscopic methods and principal analytical applications (MIR-mid infra red; FIR-far infra red).

Optical sensors in general offer substantial advantages: they are non-invasive, can detect several compounds simultaneously, and no removal of a sample from the process is necessary [6]. Figure 1 is an overview of optical spectroscopic sensors and their principal applications. Among all of the options, in bioprocess analysis research, sensors based on the near-infrared wavelength (NIR) have gained more and more importance during the last two decades. Several applications are reported in the international literature dealing with NIR technology applied to bioprocess monitoring: from the first ones of Nishinari *et al.* [7], who described on-line control of glucose concentration during enzymatic starch hydrolysis, and Cavinato *et al.* [8], who used an external optical-fiber NIR instrument to monitor the alcoholic fermentation, to the more recent one of Jiang *et al.* [9], who applied NIR to the monitoring of a solid-state fermentation process for protein production, as well as that of Lopes *et al.* [10], where NIR was used to investigate the effect of process parameters on plasmid production.

The operating principle of NIR spectroscopy is based on the absorption of radiation in the near-infrared region of the electromagnetic spectrum (700–2500 nm), by all of the organic molecules present in the sample [11]. Absorptions are related to the overtone and combination bands of the -CH, -NH and -OH fundamental molecular stretching and bending vibrations that are observed in the mid-IR region. NIR signals are generally 10–100-times weaker in intensity than the fundamental mid-IR absorption bands. However, the weakness of the absorption is actually a benefit, providing direct analysis of samples without dilution or dispersion in non-absorbing matrices used in traditional UV/Vis and mid-IR spectroscopy. Because of the nature of the spectral signals, NIR bands are also much broader than mid-IR bands and tend to be highly overlapped, especially in the case of submerged processes, such as fermentation, due to the overwhelming presence of water in the spectra [12]. The possibility of optimizing the spectra signal using chemometric tools and the use multivariate analytical techniques, such as multilinear regression (MLR), principal component analysis (PCA) or partial least squares analysis (PLSA), permit one to extract meaningful information from the complex NIR spectra and quantitatively correlate them to the concentration of the parameters of interest [13]. NIR spectroscopy is an indirect analytical technique that provides two phases before its utilization: calibration and validation. At the first step, several samples are analyzed by both a conventional method and by the NIR apparatus. The reference values are then regressed against the NIR data obtained from the spectra in order to produce, for each key parameter, a calibration regression model. Comparison between NIR predicted values and conventional methods of measurement on a new set of samples provides the basis for the validation step, carried out in order to test the robustness and the predictive capability of the calibration.

To date, the use of NIR spectroscopy as a viable alternative to traditional methods of analysis has been becoming an established option thanks to the rapid development of improved instruments and data processing techniques.

Modern NIR instruments are usually classified in terms of technology employed for wavelength selection: filters, light emitting diodes (LED), diode arrays, acousto-optical tunable filters (AOTF), dispersive optics and Fourier transformation (FT). Generally, low cost instruments, based on filters and LEDs, are useful for many dedicated lab and routine in-field applications, while instruments based on dispersive optics and sensors arrays have proven to be a robust solution when multi-wavelength spectral data for in field applications are required. AOTF- and FT-based instruments must be the choice when research, wide application spectra and calibration transference are of concern [14].

For interfacing the spectrometer with the bioprocess reactor, there are the possibilities of an *in situ* (or *in-line*) or an *ex situ* (or *on-line*) measurement approach. While for the *in situ* measurements, a fiber optic probe, which is directly immersed into the fermentation broth, is usually utilized, the *ex situ* approach can be realized by either using a flow-through cell or loop or by a reflectance probe on the glass wall of the reactor.

This paper describes the application of NIR spectroscopy in fermentation monitoring of the key biochemical parameters (substrate, metabolites and biomass concentrations), using anaerobic homolactic fermentation and aerobic heterolactic fermentation as case studies. In particular, we discuss here the potentiality of an *on-line* and *in-line* NIR instruments, the control capability in different fermentation strategies (batch, repeated fed-batch and continuous) and the matrix effects on spectra signal acquisition in the case of an immersed probe. Finally, we tested the capability of NIR to discriminate among different types of bacteria and the potentiality to transfer the calibration from one process to another, carried out in the same experimental conditions.

## 2. Experimental Section

### 2.1. Microrganisms and Cultivation Media

The strain used for homolactic fermentation was *Lactobacillus casei* DSM20011 (ATCC 393), a homofermentative *Lactobacillus* known for L-lactate production [15]. The experiments were performed in MRS broth (Merck), composed of: glucose, 100 g/L; yeast extract, 30 g/L; MgSO_4_·7H_2_O, 0.6 g/L; sodium acetate, 1 g/L; FeSO_4_·7H_2_O, 0.03 g/L; MnSO_4_·H_2_O, 0.03 g/L; KH_2_PO_4_, 0.5 g/L; K_2_HPO_4_, 0.5 g/L; pH 6.50 ± 0.01 after sterilization; temperature, 37°C.

For heterolactic fermentation, different strains, growing in the same complex glucose-based medium and in the same conditions of pH and temperature, were employed: *Staphylococcus xylosus* ES13, *Lactobacillus fermentum* ES15 and *Streptococcus thermophilus* ES17, supplied by the collection of the Department of Pharmaceutical Sciences (University of Bologna) [16].

The three microrganisms were cultured in M53 modified broth, with the following composition: glucose, 46 g/L; yeast extract, 5 g/L; bacteriological peptone, 12 g/L; MgSO_4_·7H_2_O, 0.6 g/L; FeSO_4_·7H_2_O, 0.03 g/L; MnSO_4_·H_2_O, 0.01 g/L; KH_2_PO_4_, 3 g/L; NaCl, 6 g/L; (NH_4_)_2_SO_4_, 10 g/L; group B vitamins, 5 × 10^−5^ g/L; pH 7.00 ± 0.01 after sterilization; temperature, 37°C.

All mediums were sterilized for 20 min at 120°C. Glucose was always sterilized separately and added afterward to avoid broth browning due to Maillard reactions.

The stock cultures were stored in glycerol (20%), kept at −80°C and subcultured for 24 h in 100 mL of broth before being used as inoculum for the fermenter.

### 2.2. Fermentation Equipment and Conditions

A BM PPS3 3000 fermenter (Bioindustrie mantovane, Mantova, Italy) having a working volume of 2 L was used. The bioreactor was completely computer controlled and, thus, could automatically maintain the programmed conditions of temperature, pH and air saturation (when needed). Constant stirring was maintained (120 rpm) during homolactic fermentation. Anaerobic conditions were assured by passing nitrogen gas through the headspace of the bioreactor and sparged into the culture. In heterolactic fermentation, the agitation rate (from 100 to 1000 rpm) and aeration were automatically regulated in response to oxygen concentration to a maximum of 0.75 vvm (volume per volume per minute).

At regular intervals throughout the fermentation, at the same time as spectra collection (see below), samples were withdrawn aseptically form the bioreactor and subjected to off-line analysis to determine the concentration of glucose, lactic acid, acetic acid and dry cell mass. The medium was not replaced after withdrawal, because the volume of the sample removed could be considered negligible. Glucose was the main carbon and energy source, while lactic and acetic acids were the main products of its catabolism (only lactic acid in the homofermentation, both in the heterofermentation), apart from carbon dioxide, which was not measured.

Batch, repeated fed-batch and continuous fermentation strategies were carried out. In continuous cultures, the steady state was considered to be attained when all of the parameters monitored remained constant for at least two residence times.

The microfiltration system (Bioindustrie mantovane, Italy) was composed of two tangential cross-flow units, *in situ* sterilizable with steam, containing ceramic modules (pore size of 0.2 mm) with a total filtering surface of 0.24 m^2^ and equipped with a counter-current cleaning system to prevent fouling due to biomass accumulation.

### 2.3. Off-Line Reference Assays

The biomass concentration was determined as dry weight by a membrane filtration method (Millipore^®^ cellulose acetate filter; pore size: 0.45 μm). After drying of the filters at 105 °C to a constant weight, 10 mL of the broth samples were filtered under vacuum. After washing with distilled water to remove dissolved substances, the filters were dried again to a constant weight and weighed. The net dry cell weight was obtained by subtracting the weight of the empty filters.

Glucose, lactic acid and acetic acid were determined by HPLC (Shimadzu, Japan). The isocratic condition was employed using an ion-exchange Aminex^®^ HPX-87H column (300 mm × 7.8 mm) at 25 °C with 0.005M H_2_SO_4_ as the eluent (flow 0.6 mL/min) and a refractive index detector. Before the analysis, all broth samples were centrifuged for 10 min at low temperature (5 °C) to inhibit metabolic activities.

### 2.4. NIR Spectroscopy and Data Collection

To carry out on-line and in-line experiments, two different NIR instruments were used.

For the on-line monitoring of anaerobic homolactic fermentation, an InfraAlyzer 450^®^(Bran+Luebbe, Germany) was used. The instrument was equipped with a flow cell (27 mm diameter, 0.2 mm thickness, 114 mm^3^ volume, gold-plated bottom) inserted in a closed loop through which the culture was continuously circulated by means of a peristaltic pump. The broth was pumped into the cell and then returned to the vessel with a flow rate of 40 mL/min and a sample residence time on the cell of 0.171 s. The sample was irradiated with the required NIR radiations, through a standard filter wheel (19 filters, spectral range 1445–2348 nm). It is capable of outputting results to an external computer for calibration. Part of the radiation is absorbed by the sample, whereas the remainder is reflected and collected by an integrating sphere as scattered light (reflectance mode). The cell was never rinsed during on-line operation, but the cleaning step was carried out only at the end of the fermentation when the loop was disconnected. The cell was cleaned with distilled water and ethanol, in turn. The instrument is capable of the output of data to an external computer; so for calibration, they are elaborated using UNSCRAMBLER 7.5^®^ software (CAMO, Norway).

For the preparation of calibration curves of glucose, lactic acid and cell biomass, 45 samples of broth were collected. Then, validation tests were carried out by using 30 new broth samples.

The experimental device for the in-line monitoring of heterolactic fermentation was built up by a stainless steel (AISI 316) immersion probe inserted into the bioreactor top-plate and connected to a FOSS-NIRSystem 6500^®^ spectrometer by means of a 2.5-m bundle of optic fibers. Data collection was made by a slit set in the lower part of the probe, completely immersed in the culture. Light energy is carried out from the source to the sample through the outer illumination ring of the fibers, interacts with the sample and is reflected back by a high-mirrored sapphire surface (transflectance mode). The slit thickness could be adjusted from 1 to 10 mm, but according to our experience, we have set it to 1 mm, thus providing an optical path of 2 mm. This allowed the absorbance measured in the analytical relevant part of the spectrum to remain below 4 AU, which is reported as the limit above which linearity is lost using this instrument. NIR spectra were collected from 700 to 2500 nm. In the case of the fiber optic probe, the upper limit of the spectra was 1800 nm, because up to this wavelength, the instrument sensitivity is weakened by the effect of fiber-optic attenuation.

Data were processed by means of two software packages: VISION^®^ 2.20, supplied with the instrument, and WINISI II^®^ (Foss, Denmark).

### 2.5. NIR Spectra Analysis

During fermentation runs, for each sample and at the same time as the withdrawal to off-line analysis, a single NIR spectrum of the culture was collected, stored in the PC connected with the NIR instruments and processed by means of the software packages used in the different cases.

In the on-line set of spectra, an MLR analysis was carried out.

In the case of in-line monitoring, the instruments acquired the full spectrum, and so, enhanced chemometric tools were necessary to extract analytical information from the signals. PCA was carried out on the acquired spectra in order to select redundant samples and/or outliers and to obtain the best calibration sets (Mahalanobis distance, 3σ; probability level, 95%). All spectra were then pretreated (second derivative) to enhance spectral features and reduce baseline offsets, according to the Norris method [17]. In this latter case, the extraction of information from the spectra required a PLS multivariate approach. Calibration curves were built including 80 samples in the set, and the 30 new samples were collected for the validation set.

The accuracy of the models was evaluated as the standard error of calibration (SEC), the standard error of prediction (SEP) and the root mean standard error of prediction (RMSEP) [18].

## 3. Results and Discussion

### 3.1. On-Line Monitoring of Anaerobic Homolactic Fermentation

**Figure 2.**
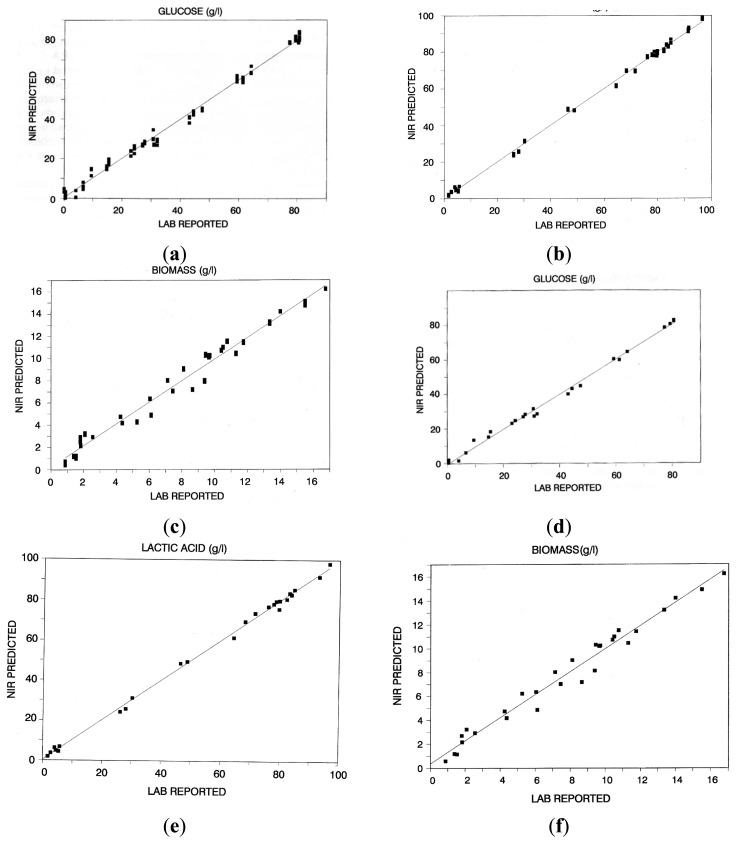
NIR calibration curves for (**a**) glucose; (**b**) lactic acid; and (**c**) cell biomass, NIR validation curves for (**d**) glucose; (**e**) lactic acid and (**f**) cell biomass. Data are expressed in g/L.

**Figure 3.**
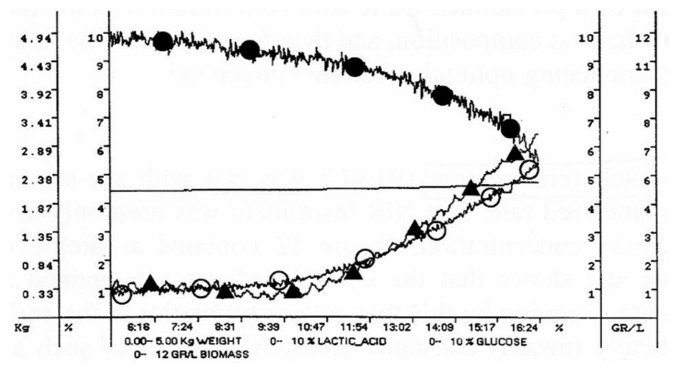
PC graphical representation of process data during batch fermentation. Data are expressed in g/L.

Calibration and validation were carried out on separate subsets of culture samples obtained from a variety of batch cultures, chosen in such a way as to cover as uniformly as possible the range of concentrations of glucose, cell biomass and lactic acid corresponding to all possible degrees of conversion of the former into the latter. Acquiring samples and spectra from real fermentation rather than on artificial mixtures of pure solutions of the individual analytes could be considered laborious and uselessly time-consuming. However, it is worthwhile to note that in this type of application, it is not possible to ignore that the instantaneous composition of a fermentation fluid is the result of the interactions between the numerous components of the matrix, whose exact composition, in any event, is almost impossible to predict. This, taken together with the highly correlated nature of most culture components (metabolite production is related to substrate consumption), shows the impossibility of simulating fermentation broth with mixtures of pure compounds, if a reliable calibration is desired. Except for the region of the spectra around 1450 and 1600 nm, completely covered by water absorption due to the first O-H overtone and O-H combination band, NIR wavelengths that presented the strongest intensity of absorption were located in the intervals 1625–1800 nm and 2200–2300 nm. These regions are dominated by characteristic absorptions corresponding to the second and first overtone of the CH bond and to the combination band of the -COOH and -OH groups, respectively. The quality of the results obtained (Figure 2) was judged to be satisfactory for the purpose of the project (Table 1). It is worth noting that the calibration of cell biomass shows a much higher dispersion of values than those for glucose and lactic acid. This can be ascribed to the low reproducibility of data generated by the off-line measurement of biomass dry weight. As mentioned above, NIR spectroscopy being a “secondary technique”, which needs a reference method to be calibrated, the errors of NIR analysis could never be lower than the errors derived from the reference method. Validation of the calibrations, carried out using external sets of batch fermentation samples, confirmed the satisfactory quality of the former. The observed differences between the NIR and lab results were evaluated by means of a paired Student's test and found not significant at the 1% level. The calibrated on-line NIR system was then used to follow batch fermentations (Figure 3) and found to represent very satisfactorily the time course of the concentration of the key parameters. In Figure 3 and in the subsequent figures, labels superimposed over solid lines represent the trend of concentrations of the key parameters obtained from reference assays.

Application of NIR *On-Line* to the Monitoring, Control and Management of Homolactic Fermentation

To reconfirm the validity of NIR measurement and to demonstrate that this technique allows real-time and automated monitoring of different fermentation processes, five different fermentation strategies were explored, each starting with a conventional batch process.

Control and management were achieved by interfacing the NIR spectrometer with the PC-based control system of the bioreactor. The system was enabled to acquire the NIR spectra of the culture at preset time intervals, to calculate the predicted analyte concentration based on the NIR validated calibrations and to relay them back to the bioreactor. Here, they were immediately displayed together with the other conventional variables, also usable by the bioreactor control system, to obtain a completely automated management of the process.

**Figure 4.**
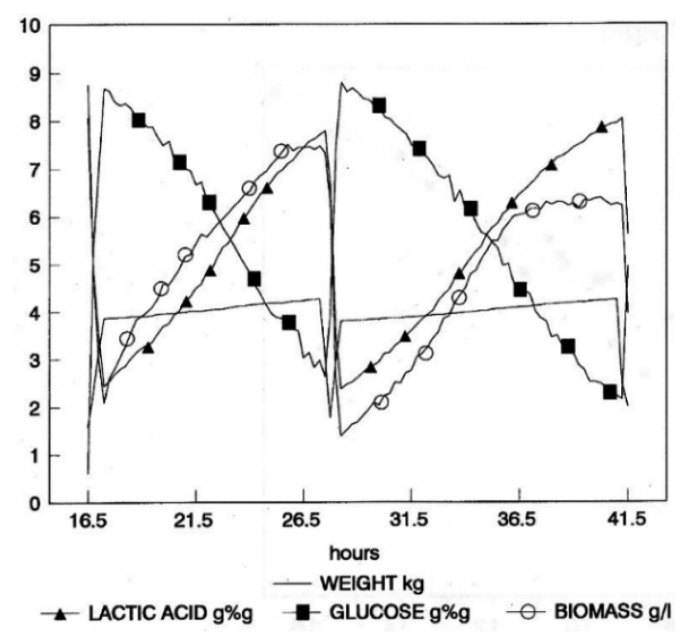
Graphical representation of process data during repeated-batch fermentation (RBC) fermentation. Concentrations of glucose and lactic acid are expressed in % (w/w) (glucose and lactic acid); concentrations of cell biomass are in g/L.

A repeated-batch fermentation (RBC) technique was adopted, where culture removal was triggered by the achievement of a pre-defined concentration of lactic acid. Culture volume was reduced to a pre-determined value, and then, fresh medium was rapidly added to reach the set cultivation volume. Culture discharge and re-fill were automatically managed by the bioreactor weight control system, based on the real-time values supplied by the NIR on-line (Figure 4). Such a simple fermentation strategy is essentially aimed at reducing the non-productive phases of the fermentation. The achievement of a pre-defined lactic acid concentration as the harvesting criterion ensures reproducible harvest composition and, therefore, reproducible downstream processing, in addition to guaranteeing optimal substrate conversion.

**Figure 5.**
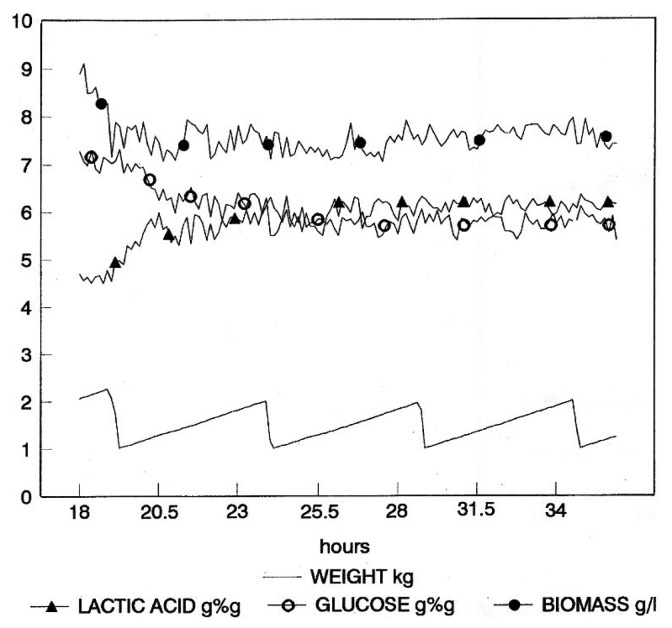
Graphical representation of process data during repeated-batch fermentation (RBC) fermentation, with preset values for initial volume, final volume and feed rate. Concentrations of glucose and lactic acid are expressed in % (w/w) (glucose and lactic acid); concentrations of cell biomass are in g/L.

Then, a conventional repeated fed-batch fermentation (RFBC) was run with pre-set values of initial volume, final volume and feed rate. The NIR instrument was used only to monitor the substrate, product and biomass concentration. Figure 5 contains a sample of three consecutively fed-batch cycles and shows that the culture had not yet attained a cycle-independent physiological state, meaning that the culture properties at the end of each cycle should tend asymptotically towards constant values. Once this state is achieved, the calculation of the productivity of the process as a function of the number of cycles becomes straightforward [19].

**Figure 6.**
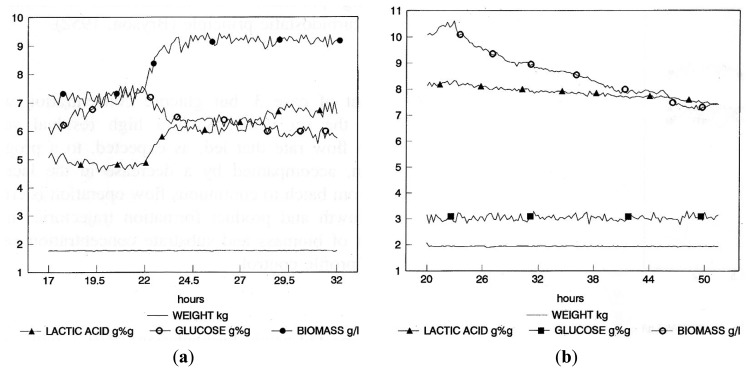
Graphical representation of process data during continuous fermentation (CF) with (**a**) biomass and (**b**) glucose as the controlled variable. Concentrations of glucose and lactic acid are expressed in % (w/w) (glucose and lactic acid); concentrations of cell biomass are in g/L.

Two continuous flow cultivations (CF) were run, in the first case with biomass concentration as the controlled variable. It was essentially a turbidostat at constant volume, where medium flow was modulated to maintain biomass concentration at a preset value. Constant volume was maintained by the bioreactor weight control system acting on the discharge pump controlled by the NIR monitoring system. Figure 6a shows the time course of an experiment where biomass concentration was first set to 7 and then to 9 g/L. The resulting medium flow rate (glucose concentration) required keeping the biomass at the set value, which was obviously lower in the second case. The latter experiment was analogous, but the glucose concentration was the controlled variable, with 30 g/L as the set value. Such a high residual substrate concentration required a high medium flow rate that led, as expected, to a progressive decrease of the biomass concentration, accompanied by a decrease in the lactic acid concentration (Figure 6b). Real-time NIR measurements of biochemical parameters have been demonstrated to be a valuable tool for technical operators and profile controls.

**Figure 7.**
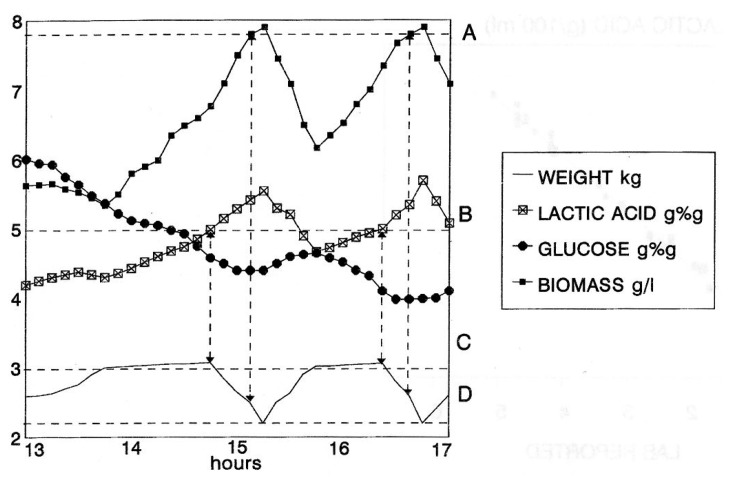
Graphical representation of process data during RBC fermentation with periodic product removal by microfiltration and periodic removal of the excess of cell biomass. Concentrations of glucose and lactic acid are expressed in % (w/w) (glucose and lactic acid); concentrations of cell biomass are in g/L.

Finally, an experiment of the completely automated management of a process, based only on the real-time NIR results, was attempted. An RBC fermentation with periodic product removal by microfiltration and periodic removal of the excess of biomass was set up (Figure 7). When a preset value of lactic acid concentration (B) was exceeded, the microfiltration module was activated. As a result, the biomass concentration increased. When the biomass threshold (A) was reached, the culture was automatically discharged until a preset volume (D) was left inside the bioreactor. At this point, automatic weight control took over to reconstitute the culture volume to the preset working level (C) by adding fresh medium. A new cycle was triggered by the lactic acid concentration reaching the (B) threshold. The net result of such a strategy is that biomass concentration is kept at the maximum workable level, thereby maximizing the rate of conversion, and lactic acid is kept at the optimal level that combines a low cost of recovery with an acceptable level of product inhibition.

### 3.2. In-Line Monitoring of Aerobic Heterolactic Fermentation

**Figure 8.**
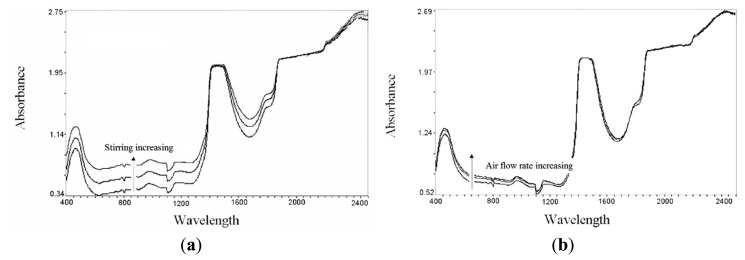
Water spectra measured with (**a**) variable stirring rate (100–1000 rpm) and (**b**) variable air flow rate (0.1–1.0 L/min).

**Figure 9.**
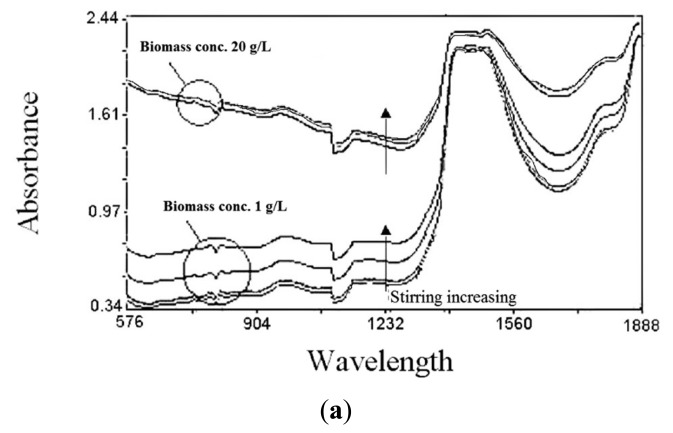

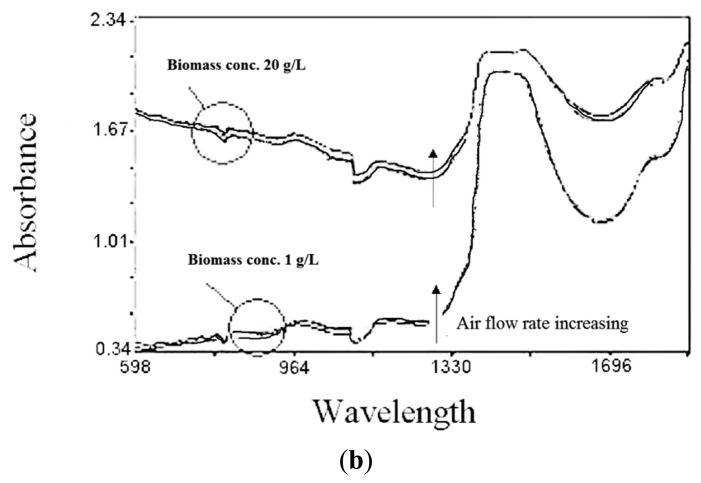
Effects on the spectra baseline of (**a**) variable stirring rate (100–1000 rpm) and (**b**) variable air flow rate (0.1–1.0 L/min), at low (1 g/L) and high (20 g/L) cell biomass concentrations.

Further research has been carried out with the aim of demonstrating the applicability of NIR spectroscopy also to the *in situ* monitoring of fermentation. The choice of aerobic bioprocess was made for investigating the possible spectral interferences deriving from heavy aeration and agitation conditions on the spectra signal. Most submerged fermentations are carried out in stirred and/or aerated conditions in order to both ensure mixing, heat transfer and promote gas-liquid oxygen exchange in aerobic processes, such as our case study. A series of preliminary runs were carried out for evaluating potential interferences of stirring and aeration upon NIR spectra. Moreover, some tests have been executed to evidence the possible presence of probe fouling. Several spectra have been collected in conditions of high biomass concentration (20 g/L) over a wide period of time (20 h). Any signal drift between NIR predictions and off-line analytical responses was found to occur with reference to fouling of the optical window, thus making it unnecessary to resort to an *in situ* cleaning system. However, a marked influence of the hydrodynamic conditions on the quality of the signal was detected. In particular, substantial baseline shifts were induced by changes in the population of air bubbles suspended in the culture. The role of agitation/aeration conditions was confirmed by recording NIR spectra of variously aerated and stirred distilled water. In relative terms, stirring speed was found to influence spectra more than the air flow rate, presumably due to its stronger effect on bubble size reduction and fluid velocity (Figure 8) [20]. The increase in the gas-liquid interfacial area is obtained through a reduction of gas bubble size, and this, in our view, may make it easier for gas bubbles to enter the gap between the probe window (1 mm throughout this work), thus altering the signal. In order to evaluate the importance of this effect under working conditions, a series of tests were carried out using culture broths containing various concentrations of microbial cells. It was observed that the strong relationship existing between cell concentration and light scattering is profoundly influenced by the culture hydrodynamics. In particular, if the gas flow rate is kept constant and the stirring speed is progressively increased, very marked baseline shifts are observed, especially at low biomass concentrations, e.g., 1 g/L dry cell mass (Figure 9a). The effect is less marked at higher cell-mass concentrations, e.g., 20 g/L, because the effect of air bubbles may be partly hidden by the influence on the spectra of light scattering due to the ever-increasing number of cells. The ambiguity of analytical response may result, not so much from spectra baseline shifts, as from its being changeable with the increase of biomass concentration. Variations in the gas flow rate at constant stirring speed were also found to influence spectral baselines, both at low and high cell-mass concentrations, but to a lesser extent than stirring speed (Figure 9b). As already pointed out, this may be induced by the fact that increasing gas flow rates produce larger numbers of bubbles, but of larger dimensions, thus decreasing the probability that they can enter the probe gap. These effects as a whole, together with changes in light scattering due to the increasing number of cells, can lead to errors in the quantitative determination of the various analytes. Consequently, to minimize these errors, whereas increasing of light scattering has been included in the calibration model as a variable, stirring speed had to be kept constant throughout the fermentation runs. If keeping a constant stirring speed was impractical, consistently with the characteristic of several bioprocess, further improvements on calibrations could be introduced by properly increasing the number of samples and including stirring-speed variations in the model, as well, as an additional source of variability in the system.

The four calibrations and validations were built on sets of samples obtained from real batch fermentations, as in the previous case. Results are reported in Table 2. The best quantitative PLS regression models was evaluated based on the squared correlation coefficient (R^2^), the standard error of calibration (SEC) and the standard error of cross-validation (SECV), which is an automatic procedure of the software to internally validate the calibration and standard error of predictions (SEP).

The best models were selected based on the minimum value of predictive residual errors sum of squares (PRESS) [21]. In a complex system, such as a fermentation matrix, a rather high number of factors was required. In our case, all four calibration models are in the range of 8 to 12.

Application of NIR *In-Line* to the Monitoring, Control and Management of Heterolactic Fermentation

The encouraging results obtained using the immersion probe prompted us to evaluate the actual applicability of in-line NIR spectroscopy to the control of real fermentation processes, using the same interfacing system used in the previous case.

This tool was used to monitor repeated batch and continuous fermentations. In a repeated batch process, at the end of the cells' exponential multiplication phase (15–20 h), defined by the exhaustion of the main carbon source (glucose), a portion of exhausted broth was drained out, and an equivalent portion of sterile fresh medium was put into the vessel in order to take the cell's growth phase up again. It was thus possible to test the predictive ability of the models (data not shown). The trends of concentration predicted by the NIR results in real time and without any operator intervention (solid line) and those achieved from the reference assay (labels) can be almost perfectly superimposed and show very satisfactory predictions of glucose, lactic and acetic acids and biomass concentrations.

**Figure 10.**
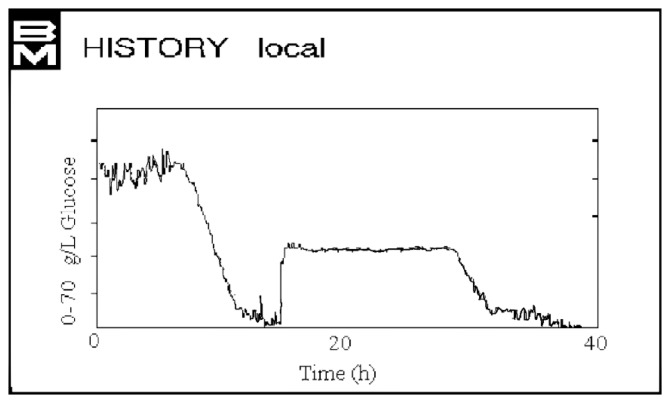
Graphical representation of NIR predicted results for the *S. xylosus* ES13 continuous fermentation processes (preset glucose concentration value: 35 g/L).

Then, a continuous culture (chemostat) where glucose was the controlled variable (35 g/L), was set up. Figure 10 shows the trend of glucose concentration, kept constant for 24 h, with the medium flow rate as the control variable based on the NIR prediction of glucose concentration inside the bioreactor.

### 3.3. NIR Capability to Discriminate among Differently Shaped Bacteria

A further aim of this investigation was to evaluate whether NIR was able to discriminate among morphologically different bacteria growing in the same media, in similar fermentation conditions. Using suitable chemometric tools, it was investigated whether, in the NIR spectra, regions or particular wavelengths clearly attributable to one bacteria could be identified. Several studies have been done on the capacity of NIR to discriminate among microbial strains belonging to different species (*i.e.*, bacteria from yeast), thanks to the specific “biochemical fingerprint” of cell membranes [22,23].

**Figure 11.**
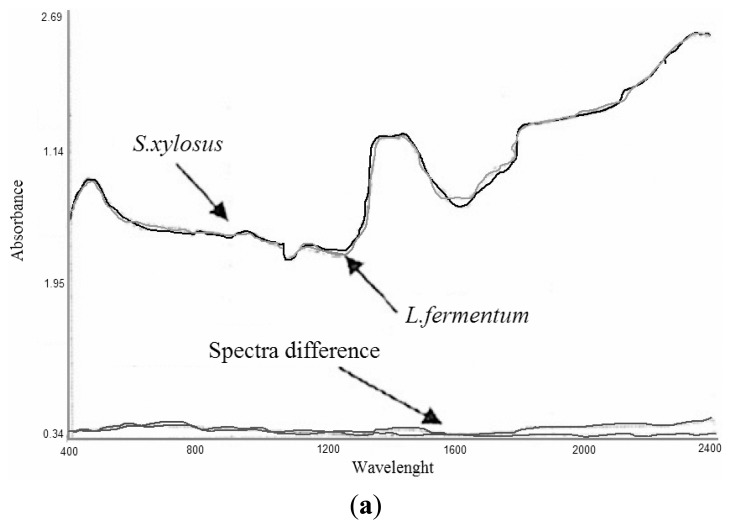

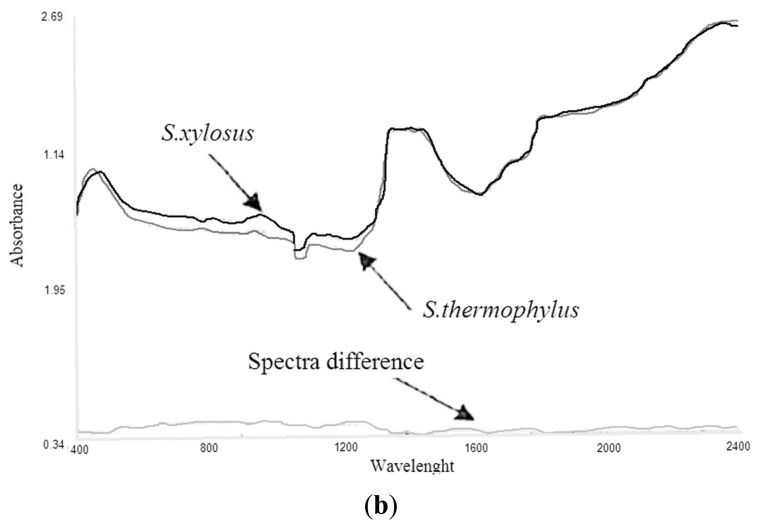
Two by two differences between mean spectra of the cultural broth of *S. xylosus* ES13, *L. fermentum* ES15 and *S. thermophilus* ES17.

In addition to *S. xylosus* ES13, which forms typical clusters of cells, two other microrganisms, the rod shaped *L. fermentum* ES15 and the spherical *S. thermophilus* ES17 were chosen, because, although microaerophilic, they could grow in the same cultural medium and similar hydrodynamic conditions. Several batch fermentations were carried out to collect spectral information representative enough for both microrganisms. The mean spectra were calculated for each data set and subtracted two by two, showing the lack of any significant spectral differences specifically assignable to grape, rod or spherical shapes (Figure 11). The evidence was that NIR was not able to discriminate among the different bacteria, probably because if any distinctive spectral features existed, they were completely covered by the predominant water absorption. This notwithstanding, these results laid the ground for the hypothesis of using the same calibration models in processes with different microrganisms, reducing the time necessary for the laborious procedure of calibration building. Therefore, calibration models developed for *S. xylosus* ES13 could be successfully applied to predict the same parameters throughout the fermentation of *L. fermentum* ES15 and *S. thermophilus* ES17. The results obtained are reported in Tables 3 and 4, respectively. Using models previously constructed for glucose, cell biomass and lactic and acetic acids, only another 20 samples were collected for the external validation. The data presented were considered satisfactory, in terms of correlation and SEP, for all four variables. This means that, for equal hydrodynamic conditions and matrix composition, for each constituent, only a rapid validation was needed to evaluate the effective prediction ability with the new system.

## 4. Conclusions

The results obtained in this study clearly demonstrate that direct on-line and in-line NIR spectroscopy can be used for the real-time monitoring and control of the key biochemical parameters in a bioprocess. Interfacing to a suitable bioreactor control system allows a number of different fermentation strategies to be implemented successfully. While a number of technical aspects, *i.e.*, the use of *in situ* probes *vs.* external circulation loops, probe geometry, *etc.*, need to be addressed for the specific process from time to time, a generalized application potential is ready to be exploited in production processes, as well as in process development and optimization. The quantitative results were considered highly satisfactory, in terms of both accuracy and precision, emphasizing that the spectroscopy-based approach to process monitoring was valid, even when applied to multicomponent time-variable matrices, typical of the bioprocess.

## Figures and Tables

**Table 1.** Calibration and external validation results for the data sets of *L. casei*, by MLR regression. RMSEC, the root mean standard error of calibration; RMSEP, the root mean standard error of prediction.

	**Calibration**			**External Validation**		
	
	**Range (g/L)**	**R^2^**	**RMSEC (g/L)**	**Range (g/L)**	**R^2^**	**RMSEP (g/L)**
**Glucose**	0–87	0.9956	4.76	0–81	0.9899	5.21
**Lactic acid**	0–98	0.9952	3.15	0.98	0.9900	3.87
**Cell biomass**	0–16	0.9901	1.98	0–16	0.9856	2.55

**Table 2.** Calibration, cross-validation and external validation results for the data sets of *S. xylosus* ES13, by PLS regression. SEP values were calculated as corrected for bias. SECV, standard error of cross-validation.

	**Range (g/L)**	**Calibration**			**External Validation**	

		**R^2^**	**SEC (g/L)**	**SECV (g/L)**	**R^2^**	**SEP (g/L)**
**Glucose**	0–58	0.9708	3.01	3.34	0.9710	2.61
**Lactic acid**	0–23	0.9381	1.50	1.66	0.9393	1.36
**Acetic acid**	0–19	0.9573	0.95	1.05	0.9010	0.62
**Cell biomass**	0–16	0.9506	0.91	1.15	0.9535	0.85

**Table 3.** External validation results of *S. xylosus* ES13 models applied to *L. fermentum* ES15.

	**Range (g/L)**	**R^2^**	**SEP (g/L)**
**Glucose**	0–56	0.9651	1.83
**Lactic acid**	0–22	0.8926	1.64
**Acetic acid**	0–15	0.8136	0.51
**Cell biomass**	0–16	0.9358	0.95

**Table 4.** External validation results of *S. xylosus* ES13 models applied to *S. thermophilus* ES17.

	**Range (g/L)**	**R^2^**	**SEP (g/L)**
**Glucose**	0–50	0.9256	3.6
**Lactic acid**	0–24	0.9547	1.5
**Acetic acid**	0–8	0.9655	0.7
**Cell biomass**	0–12	0.9188	0.9
